# Exploring Narrative and Coping Strategies of Osteosarcoma Survivors in China: A Qualitative Study

**DOI:** 10.1002/cnr2.70170

**Published:** 2025-03-04

**Authors:** Ziqi Peng, Suet Lin Hung, Kwok Kin Fung

**Affiliations:** ^1^ Department of Social Work Hong Kong Baptist University Hong Kong SAR China

**Keywords:** coping strategies, narrative inquiry, osteosarcoma, psycho‐oncology, survivorship

## Abstract

**Background:**

The existing studies have pointed out that cancer survivorship experiences significantly contribute to a comprehensive understanding of cancer survivors and insights into oncological care development. However, little is known about the long‐term survivorship experiences of osteosarcoma survivors within the Chinese context.

**Objective:**

This study aims to explore osteosarcoma survivorship and coping strategies to enrich our understanding of the unique challenges and psychological needs of this population.

**Methods:**

A qualitative narrative inquiry was used to provide a comprehensive understanding of survivors' illness narratives and coping strategies during osteosarcoma survivorship. Twelve osteosarcoma survivors were recruited using purposeful sampling. The data were collected through narrative inquiry interviews, which were audio‐recorded and transcribed. A thematic narrative analysis is used to interpret the narrative inquiry data.

**Results:**

Three themes of illness narratives were identified, including “No one knows: chaos,” “Rethinking: restitution,” and “Restarting and retelling: quest.” Coping strategies encompassed in the subthemes indicated the importance of archive efforts of osteosarcoma survivors in questing post‐traumatic identity, values, and reconnecting to the community. Alternative narratives that emerged beyond the illness narrative framework highlighted contextual‐specific insights.

**Conclusion:**

This study demonstrates that a narrative approach provides an alternative perspective for exploring the cancer survivorship experience. The findings underscore the evolving nature of illness narratives, highlighting the long‐term need for ongoing psychological support for osteosarcoma survivors. Moreover, the study highlighted a comprehensive understanding of osteosarcoma survivorship within the Chinese context. Specifically, it emphasizes (1) the rethinking of current medical discourse and psycho‐oncology practice within the Chinese context and (2) the narrative empowerment of bone cancer survivors. These findings have significant implications for expanding exploration into the nuanced experiences and identification of barriers to the integration of medical and psychological support among Chinese cancer survivors.

## 1 Introduction

While existing literature has examined cancer patients' experiences and coping mechanisms, emphasizing the significance of mental health care, self‐disclosure, quality of life (QoL), and post‐traumatic growth, there remains a notable gap in understanding the long‐term experiences, illness narratives, and coping strategies specific to osteosarcoma survivors [1, 2, 3, 4, 5, 6, 7, 8, 9, 10, 11]. Osteosarcoma is the most commonly prevalent in youth and adolescents aged 10–20 years old [1, 12, 13]. In this stage, the survivor is still under a significant stage for dealing with psychological development. However, osteosarcoma patients not only share the same confusion as their peers but also struggle with the side effects of chemotherapy (nausea, constant tiredness, etc.), fear of relapse and metastases [10, 14, 15], anxiety, depression symptoms [7, 10], and identity disruption [2, 3]. Additionally, osteosarcoma has profound, multifaceted, and long‐term impacts on the life trajectories of youth and adolescent survivors. Cancer diagnosis frequently interrupts educational participation in cancer survivors and limits their pursuit of academic achievements [16]. Specifically, previous studies have documented how cancer treatment correlates with the experiences of lower school attendance in academic and physical activities, peer exclusion, and appearance‐based stigmatization among survivors [16]. Osteosarcoma survivors additionally experience restricted limb mobility, which further impacts their participation in these activities. Moreover, current integrative approaches across family, school, and medical settings remain insufficient in addressing these complex challenges [16]. The examination of illness narratives and coping strategies among osteosarcoma survivors provides significant and valuable insights for professionals seeking to understand survivors' needs and challenges throughout their recovery journey. These narratives illuminate how survivors cope with physical body changes affecting decreased QoL and negative body images [9, 17] and resume educational participation [16]. These findings have significant implications for support interventions provided by professionals serving this population.

In the context of mainland China and Hong Kong, we have identified dilemmas that limited the psycho‐oncology practices to addressing the psychological needs of cancer survivors. While Chinese psycho‐oncology practices are emerging, a significant challenge lies in the dearth of appropriately trained professionals to effectively fulfill these roles [18, 19]. Specifically, the dominant medical perspective and underdeveloped knowledge of psycho‐oncology in bone cancer serve as potential rationales [18, 20, 21, 22, 23, 24]. First, studies in mainland China and Hong Kong show the post‐traumatic follow‐up of osteosarcoma survivors predominantly focused on medical issues or treatment outcomes [21, 22, 25]. The dominant medical discourse and pharmacological interventions [26, 27] might lead to the overlook psychological needs of cancer survivors [28]. Secondly, limited knowledge of psychological care within the context of osteosarcoma hinders the comprehensive implementation of psychological interventions for patients and survivors [21, 22, 25]. We recognize Chinese nurses' responsibility to address the psychological needs of cancer survivors, but they lack relevant skills and knowledge within the nursing profession [23]. Furthermore, the existing separation model between medical and psychological practices hinders the integration of psycho‐oncology interventions within cancer treatment, potentially limiting survivors' access to comprehensive support [24, 29, 30]. As X. Liu et al. [25] pointed out, “There are no intervention studies on drawing psychotherapy for children and adolescents with osteosarcoma.” Given these existing challenges, empirical evidence indicates that over one‐third of Chinese bone cancer survivors experienced psychological issues relevant to dissatisfied body image and depressive, anxiety, and avoidant personality symptoms [31].

This study focused on the narrative of osteosarcoma survivors within the Chinese context. It aims to examine the osteosarcoma survivorship experience and coping strategies, thereby enriching our understanding of the unique challenges and psychological needs of this population. From this perspective, we aim to contribute to the growing body of literature that advocates for a multidisciplinary narrative approach in supporting osteosarcoma survivors within the specific context of China [10, 13, 32, 33, 34, 35]. A narrative perspective on the experiences of osteosarcoma survivors facilitates the deconstruction of dichotomous discourse that separates cancer patients from the meanings of survivorship [33, 34]. Specifically, this approach can critically examine existing models of medical and psychological practices in the Chinese context. By enriching the understanding of long‐term post‐traumatic growth in osteosarcoma survivors, this perspective underscores the need for broader integration of psychological practices and medical treatment [16, 36]. Ultimately, this integrated approach can better address the multifaceted psychological needs of osteosarcoma survivors and potentially other cancer survivors within the Chinese context.

### 1.1 Coping Strategies Relevant to People With Cancer

Coping encompasses a dynamic process of thoughts and behaviors that individuals employ to manage physical or psychological challenges [10, 37].

Coping strategies for people with osteosarcoma are frequently narrowed to their skill in managing physical function limitations and psychological aspects such as depressive symptoms caused by chemotherapy, surgery, amputation, and pain [8, 13, 26, 38]. Common coping strategies employed by cancer survivors include denial, behavioral disengagement, venting, self‐blame for ongoing chemotherapy‐related anxiety and depression [7], seeking social support, positive affectivity for post‐traumatic growth [6], and emotional adaptive coping, like optimism and reflection during illness for emotional distress in the cancer journey [39]. The study of coping strategies not only provides valuable insights for caregivers in developing targeted interventions to address the specific challenges faced by osteosarcoma survivors but also underscores the unique and multifaceted nature of post‐traumatic growth within the survivorship experience [6, 7, 39].

### 1.2 Illness Narrative and Cancer Survivor

Illness narratives offer a valuable approach to understanding the connection between illness and life events, including the process of identity construction and negotiation [40, 41]. These narratives serve as a forum for survivors to articulate and explore their lived experiences, challenges, and recovery journeys [40, 41]. Building upon Frank's [42] framework, illness narratives can be categorized as chaos narratives (e.g., vulnerability, futility, and impotence), restitution narratives (e.g., the process of reobtain health), and quest narratives (e.g., understanding of the meaning of the disease) (see Table 1).

**TABLE 1:** Illustration of three types of illness narratives.

Chaos	Restitution	Quest
The chaos narratives reveal vulnerability, futility, and impotence. “Life never getting better.” [42]	The restitution narratives are about the process of reobtain health both physically and mentally. “Yesterday I was healthy, today I'm sick, but tomorrow I'll be healthy again.” [42]	The quest narrative is the recognition and understanding of the insight and meaning of the disease. “Illness as a journey.” [42]

These narrative frameworks depict a process from illness‐related unpredictability chaos to the integration of both past learning and cultivating new understandings to navigate and overcome their illness [40, 42, 43]. Importantly, illness narratives contain valuable insights and experiences that illuminate the journey of cancer survivors as they strive to regain control of life and diminish the sense of marginalization [44, 45].

## 2 Methods

### 2.1 Study Design

This study was designed as a qualitative study. Participants were recruited through purposive sampling using survivor community advertising, online advertising, and snowball strategies between survivor networks. Participants match the inclusion criteria as follows: (1) experience osteosarcoma, (2) survive at least 1 year, (3) participants are able to speak Cantonese or Mandarin, and (4) willingness to reflect upon their experiences. The first author introduced the purpose of the study to eligible participants identified in community, online, and peer networking. There are parts of the participants who voluntarily provided photos relevant to amputation or other cancer treatment as an identifier for recruitment. Participant information is confidential throughout the study and analysis, and the participants' names are pseudonyms. Each eligible participant received a cash incentive after completing the interviews.

### 2.2 Data Collection and Analysis

Narrative inquiry interviews were conducted by the first author and a research assistant in order to collect osteosarcoma survivorship narratives from participants. This interview approach is insight from the narrative inquiry approach [46]. Each participant was conducted in one to two interviews and audio‐recorded, with interviews ranging from around 60–90 min. Extra interviews were requested for some participants due to limited time arrangements in the first section. When conducting interviews, the investigator used a private room or online meeting software to ensure a comfortable and confidential environment and that the narrative inquiry interview went smoothly. Initially, the interviewer collected survivors' demographic characteristics. The interview focused on the survivor's experience and coping narrative construction process associated with osteosarcoma survival. The sample of questions asked was, “How did you move from treatment to recovery?” Follow‐up questions were employed based on the interviewee's response. All the interviews were conducted in Cantonese or Mandarin.

Interviews were transcribed verbatim by the investigators and research assistants. The transcripts were cross‐checked with the recordings to ensure the accuracy of the information. After data processing, transcripts were uploaded to MAXQDA (version 24.4.1) and analyzed using thematic narrative analysis [47]. We used Frank's [42] illness narrative framework as a guide in analyses. The first author was responsible for the initial coding and analysis, and the other authors were primarily oriented toward revising and reviewing the result framework generation. The authors validated the framework by comparing the narrative and cross‐checking coding. The final extracted coding reached a consensus by the authors that the data is considered saturated. The investigators' perspectives also influence the interpretation of the data [46, 48]. To ensure the quality of the report, the COREQ (COnsolidated criteria for REporting Qualitative research) checklist was used.

We acknowledged the first author's perspective may have been influenced by personal experience as a cancer survivor. This personal experience may have subtly shaped the interpretation of the data, potentially emphasizing alternative narratives that extend beyond the clinical experience of cancer to encompass personal growth and social empowerment.

## 3 Results

### 3.1 Participants

This study included 12 participants, all osteosarcoma survivors residing in various regions of China. Table 2 presents the demographic profile of these participants. The mean age of the participants was 22.8 years. The majority underwent limb salvage treatment (*n* = 9) and had tumors primarily located in their lower limbs (*n* = 9). The spatial distribution of participants mainly aggregates in southern China (*n* = 10), including dominant numbers in Guangdong (*n* = 3) and Hunan (*n* = 2). Importantly, none of the survivors reported receiving any systematic psychological counseling during their treatment and recovery process. Additionally, four participants (not included in these 12 participants) withdrew from the study after reviewing the interview questions, citing their unwillingness to disclose personal experiences.

**TABLE 2:** The demographic profile of participants.

(*n* = 12)	
Gender	
Male	6
Female	6
Mean age (range)	22.8 (13–34)
Mean years of survival (range)	7.8 (1–26)
Employment status	
Full‐time employment	6
Student (college and secondary level)	6
Treatment	
Limb salvage	9
Amputation	3
Occurrence body part	
Upper limbs	3
Lower limbs	9
Regions (Province)	
Beijing^a^, China	1
Guizhou, China	1
Guangdong, China	3
Henan, China	1
Hong Kong SAR, China	1
Hunan, China	2
Jiangsu, China	1
Jiangxi, China	1
Shanghai^a^, China	1
Received counseling	
Reported no prior experience	12

^a^ Province‐level municipality city in China.

### 3.2 Story of Participants

A total of 12 participants narrated their journey with osteosarcoma, the content encompassing valuable experiences from diagnosis to recovery. Most of the participants reported strange physical pain as an altered signal before the medical diagnosis. The shock of diagnosis concomitant with life disruption for years was identified as time stealing by cancer. These embodied reflections generate them to value day‐to‐day life after treatment and focus on the re‐engagement of relationships with family, friends, and significant others. Participants recognized that many aspects of living with cancer were beyond their control. Through their experience with medical treatment, such as chemotherapy, they disclosed the unforgettable physical hardship of this journey.

The identified themes presented evidence from the participants' narratives on osteosarcoma. The findings related to four themes. The themes refer to time steeled by cancer (*n* = 9), perceived changes in body functioning (*n* = 9), prediagnosis, strange pain, and shock by medical diagnosis (*n* = 8), and the journey of treatment (*n* = 8). Table 3 indicates the common narrative plots among osteosarcoma survivors.

**TABLE 3:** Themes of participant's survival stories.

Themes	Selected sample
Time steeled by cancer (*n* = 9)	“The cancer caused me to delay my study progress for 3 years” (Participant 1)
	“I only studied for one and a half years during my whole middle school time” (Participant 2)
Perceived changes in body functioning (*n* = 9)	“Now that my wheelchair and crutches have replaced my legs” (Participant 5)
	“After the first surgery, I had no money to continue the joint lengthening surgery, which resulted in a leg length discrepancy” (Participant 7)
Prediagnosis, strange pain, and shock by medical diagnosis (*n* = 8)	“I fell while playing badminton, and my legs suffered severe pain. Many hospitals misdiagnosed me” (Participant 12)
	“My shoulder continuously feels pain. I thought this (osteosarcoma) was definitely a misdiagnosis at that moment” (Participant 11)
The journey of treatment (*n* = 8)	“My first chemotherapy reaction was particularly severe. I had stomach pain for three days and couldn't eat” (Participant 8)
	“My emotions can be said to be constantly collapsing and then reforming” (Participant 10)

### 3.3 Survivorship Narrative and Coping Strategies

The presentation of results is organized according to Frank's [42] illness narrative frameworks (see Table 1). This framework was utilized to analyze the narratives of osteosarcoma survivors, identifying key coping strategies associated with each narrative type. The analysis sought to understand how survivors navigated the initial chaos of their illness experiences and ultimately achieved a sense of identity reconstruction.

#### 3.3.1 Themes One “No One Knows”: Chaos Narratives and Relating Coping

“No one knows” represents the chaotic narrative of osteosarcoma survivor, crystallized by suddenly interpreting their life story as cancer and turning to a long medical treatment and hospitalization. All the participants (*n* = 12) reported the chaos narrative: “No one knows,” the chaos narrative included the description of struggling with side effects of chemotherapy, pain, and loss of body functions. Table 4 demonstrates the detailed chaos narrative by participants.

**TABLE 4:** Chaos narratives.

Chaos narratives (*n* = 12)	Selected sample
	“Each round of chemotherapy limited my mobility. When I needed my father to carry me up the stairs to get home. I felt like my whole world collapsed in an instant… I feel helpless, everything is unknown… I am very scared” (Participant 5)
	“Despair is around me, the fear, pain, being unable to walk, and taking my year off from school… I broke down countless times, shed tears, everything is unknown” (Participant 9)
	“I always felt scared when I was alone. I always ask the question, ‘How long can I live?’ but no one can give me a response” (Participant 11)
	“The feeling is like a sword hanging over my head, and I don't know when it will fall” (Participant 12)
	“My parents didn't tell me how serious the illness was…when I realized amputation was done, I was completely desperate and felt that my heart and the leg were dead together.” (Participant 3)
	“I wanted to kill myself at that point, but I couldn't even do it if I didn't have the power; I don't know how to end my life; the hospital equipment was like tying me to the bed” (Participant 4)

The physical symptoms related to treatment further cause emotional stress for them and last for a long period in cancer treatment, even longer. There is a certain response to answering how long cancer takes from a survivor's life and what the consequences are. The finding is a “whirlpool” assembly of misery, disappointments, and setbacks [49]. A series of scary and unexpected traumas and pains led to another, with life appearing to go nowhere for them. While there are efforts to support the survivor to avoid further collapse, their experiences were shaped away from the socially dominant trajectory. Specifically, they were disconnected from friends and school life. Moreover, two participants narrate their serious suicidal thoughts during this period. These narratives unearth the most helpless and vulnerable moments of their cancer survivorship, which indicates a crucial stage needed for external supportive care.

Regarding the coping strategies within the chaos narrative, the findings show the participants are not passive against the struggle of medical treatment. There are coping strategies identified in their narrative. The result included two themes of coping, including seeking social support (*n* = 9) and letting the time flow (*n* = 4). For example, the survivor seeking peer support in a patient group, “We have a group (peer patients), and they told me that they all go through these processes and encourage me” (Participant 10). Table 5 demonstrates how the participants interpret their coping strategies relating to chaos narratives.

**TABLE 5:** Chaos narratives related coping.

Chaos narratives related coping	Selected sample
Seeking social support (*n* = 9)	“What I remember most clearly is that a friend loved painting. She drew lovely pictures on my only remaining limb. It is healing and makes me felt seemed to hate that leg no longer so much” (Participant 3)
	“We have a group (peer patients), and they told me that they all go through these processes and encourage me” (Participant 10)
	“The company and encouragement of family and friends are very powerful” (Participant 11)
	“When I had my first surgery, my best friend, who had already settled abroad, flew back and stayed with me…” (Participant 5)
Letting the time flow (*n* = 4)	“I guess playing mobile games is the only way to forget the pain of chemotherapy” (Participant 12)
	“I must let time flow away, such as playing games and watching TV…and sleep makes time flow quickly” (Participant 4)

Social support from the surroundings, especially family members, is significantly supported by osteosarcoma survivors who have gone through the hardship of treatment. However, identified coping shows that the strategies are not necessarily optimistic in dealing with cancer‐caused chaos. These findings suggest a crucial need for professionals to proactively identify and assess risks for psychological issues in osteosarcoma survivors, enabling the timely implementation of early interventions to prevent further exacerbation of symptoms. The results underscore the critical role of professionals in both preventing and optimizing the quality of psychological care.

#### 3.3.2 Theme Two “Rethinking”: Restitution Narrative and Relating Coping

The restitution narrative type comprises the process of reclaiming one's former state of health [42]. The findings of the participant narrative (*n* = 9) regarding the restitution narrative are considered a turning point and start a step forward from the chaos narrative (see Table 6 for the details of the participants' restitution narrative). The results pointed out that the movement of survivors recognized that the cancer was not limited to their lives and “regaining” their bodies. It is also a process by which the survivors can perceive hope of being healthy again [42, 49]. However, the analysis revealed narratives characterized by anxieties and feelings of inferiority experienced by survivors upon reconnecting to the community. These concerns may stem from an awareness of bodily changes and limitations in physical mobility. These findings indicate potential stigma and body image concerns that require intervention during the restitution period [31, 50].

**TABLE 6:** Restitution narratives.

Restitution narratives (*n* = 9)	Selected sample
	“After the amputation, my left foot touched the floor for the first time. The feeling of holding the bed railing with my hands was new and novel, like a baby learning to walk. I stood up, I finally stood up again!!! This time, my ‘wound’ finally healed” (Participant 5)
	“I was able to throw my crutches away, live like my peers, and go to school, but sometimes, I still felt inferior” (Participant 9)
	“Walking independently again is a new beginning” (Participant 6)
	“I no longer need a wheelchair, and my life has finally changed” (Participant 8)

The restitution narratives frequently highlighted “sparkling moments” instances survivor recognized and celebrated their capacity to reposition their life. The following analysis identified key coping strategies relevant to restitutive narratives, revealing a key theme of rethinking that encompassed the reevaluation of self‐identity, values, and beliefs (*n* = 9). Table 7 demonstrates the details of participants' coping strategies relating to the restitution narrative, such as looking forward back to school as a new starting life. These strategies involved strengthening interpersonal relationships, networking, and exploring personal values.

**TABLE 7:** Restitution narratives relating to coping.

Restitution narratives related coping	Selected sample
Rethinking of self‐identity, values, and beliefs (*n* = 9)	“I look forward to starting over, going back to school to take ‘Gao Kao’, and start a new life. Studying and playing with my classmates and get to a university soon” (Participant 8)
	“I started to think about being a volunteer to support the patients” (Participant 6)
	“After I adapted my prosthesis, I started to think about living in a larger city. It might have given me a friendly environment in which the community accepted me” (Participant 3)
	“I decided to wait until I recovered, learn to make choices, relax sometimes, and work less intensively” (Participant 11)
	“My family's love and care made me confirm my values of filial, family‐oriented, and responsibility” (Participant 1)

#### 3.3.3 Theme Three “Restarting and Retelling”: Quest Narratives and Relating Coping

The quest narrative is significant in that it establishes a new identity stage for survivors and redefines cancer as a part of their lives. This narrative involves externalizing the influences of cancer on their lives [42]. The participants (*n* = 7) showed the pattern of quest narrative, revealing meaning and insights from their osteosarcoma experience. Table 8 demonstrates participants' quest narrative, indicating they externalized the impacts of cancer. This signifies a successful transformation that survivor utilize their agency in transforming the negative emotional and psychological impacts of osteosarcoma. By reframing osteosarcoma as not a “problem,” participants generated a willingness to help others who may have had similar distress. We echoing Frank [42] argue, “A person who experiences deep suffering becomes the compassionate being who vows to return to earth to share her enlightenment with others.” This finding highlights the potential for peer support for osteosarcoma survivors and underscores the importance of cultivating a supportive environment for all individuals impacted by cancer.

**TABLE 8:** Quest narratives.

Quest narratives (*n* = 7)	Selected sample
	“Osteosarcoma is only a part of my childhood life. Indeed, the cancer makes me make an effort to treat people with sincerity and contribute to charity” (Participant 1)
	“I believe I have the ability to take control of the impact this osteosarcoma has had on my life. I will continuously take challenges to pursue worthwhile and meaningful things in my limited life” (Participant 4)
	“It is my rebirth; don't hesitate for the future, and never give up on finding meaning in life” (Participant 5)
	“It is only part of my background information, now I am enjoying freedom” (Participant 3)

Quest narratives most prominently articulate the transformative voices of osteosarcoma survivors. The identified coping strategies employed by these participants demonstrated their active actions in achieving personal and social commitment. Table 9 shows participants' coping strategies that commit to their quest narrative. Three key themes of coping strategies indicate the survivor's actions and commitment to their post‐cancer life journey: committing to values and giving actions (*n* = 6), voicing up the needs (*n* = 5), and reconnecting to the community (*n* = 4). These themes demonstrate the common needs of osteosarcoma survivors in starting their post‐cancer journey.

**TABLE 9:** Quest narratives relating to coping.

Quest narratives related coping	Selected sample
Committing to values and giving actions (*n* = 6)	“I chose to register the donation list of my organs and bodies. I hope to help more people when my body no longer belongs to me” (Participant 5)
	“I received an invitation to return to the hospital to share my experiences with current patients and encourage them” (Participant 6)
	“My boss was proud of me for coming here (larger city) alone, which also gave me more confidence” (Participant 3)
Voicing up the needs (*n* = 5)	“There is still a long way to go for in Chinese oncology social support for osteosarcoma survivors mental recovery. I hope everyone will pay more attention to this population so that more patients can be helped” (Participant 1)
	“I hope patients can receive more allowance for treatment and living”; “This disease cannot be cured without money” (Participants 2, 5, and 7)
Reconnecting to the community (*n* = 4)	“When I was looking for jobs, I had to try to call the director to ‘introduce’ my experience with cancer…they offered me directly. Only proud with my experience makes me not derailed society by cancer” (Participant 5)
	“Becoming a photographer makes me more outgoing, and I am happy to get more support, reflection, and inspiration from the people around me!” (Participant 4)

### 3.4 Illness Narrative Occurrence Process

Illness narratives and cancer survivorship experience highlight the entwined fluidity process experienced in illness, coping, and personal growth [51]. Table 10 is the overview of participants' occurrence of illness narrative, the matrix informed by Ray et al. [52].

**TABLE 10:** Illness narrative occurrence matrix.

Participants (*n* = 12)	Chaos narrative	Restitution narrative	Quest narrative	Years of survivorship^a^
1	✔	✔	✔	26
2	✔	✘	✔	20
3	✔	✔	✔	15
4	✔	✔	✔	14
5	✔	✔	✔	5
6	✔	✔	✔	4
7	✔	✘	✘	3
8	✔	✔	✘	2
9	✔	✔	✘	2
10	✔	✘	✘	1
11	✔	✔	✔	1
12	✔	✔	✘	1

^a^ Survivorship duration was calculated from the initiation of medical treatment.

This study recruited survivors from a range of survivorship periods between 1 and 26 years. This implies that the results vastly present differences in how these survivors form their illness narratives. Specifically, we identified the pattern that short‐period survivorship might associate with less generalized restitution and quest narrative. Thus, it means not all cancer survivors can be presented with three types of illness narrative [43]. From this perspective, we reflect on the meaning of time among osteosarcoma survivors. And raised a question that why do they have a chronological change in narrative? As Plage and Kirby [53] argue, the time configuration of cancer patients is nuanced by the perception of what is important and priorities to them. Osteosarcoma survivor experiences might be consistent with advanced cancer patients; they experience “time lost,” and their identity was disturbed by cancer [53]. The lower survivorship period might hide an issue that survivors still “surrender” in the chaos narrative rather than quest for the meaning of cancer. This argument reflected on the findings that survivors with longer survivorship have more identified narration chasing their self‐identity.

Further, this study acknowledged that illness narrative occurrence is not a linear progression; cancer survivors can shift back and forth between narratives [42]. This reflection raises questions about meaning‐making processes that evolve throughout the cancer survivorship journey. While Frank's framework underscores individual experiences for understanding illness, the process of evolving cancer survivorship might be overlooked. The development of cancer survivorship meaning emerges as a confluence between cancer experience and self‐identity construction, generating distinct identity formations for each survivor [11]. This diversity manifests in participants' varied self‐identity descriptions, including “strong woman” (Participant 11), “lucky person” (Participant 5), or “from a dancer to a wage salve” (Participant 1). While these findings may extend beyond illness narrative frameworks, they provide valuable insights into the nuanced lived experiences of cancer survivors [11].

## 4 Discussion

This qualitative study explored the Chinese osteosarcoma survivor's narrative and coping strategies in Frank's [42] illness narrative framework (see Table 1). The findings highlighted key themes including concerns, vulnerability, and coping strategies from osteosarcoma survivorship narratives. These findings were supported by distinct narrative trajectories, progressing from initial chaos to eventual restitution and, in some cases, a transformative quest narrative. In the chaos narrative, participants point out their experiences of suffering, including chemotherapy, pain, and loss of body functions, which is consistent with previous research [17, 31]. Passive coping strategies were prevalent during this stage. The restitution narrative, characterized by a turning point and emergence of sparkling moments, provided an opportunity for survivors to regain their body and leave the medical environment. It provides them a space to rethink their identity and values. These findings underscore the importance of not only providing a psychological evaluation to osteosarcoma and bone cancer patients and survivors [7] but also actively supporting documentation of sparkling moments in the osteosarcoma experience. In the quest narratives, participants demonstrated a profound understanding of the cancer experience, generating new meanings and reaffirming their future values and actions. This aligns with the literature on the cancer coping process that emphasizes reinterpretation in the face of adversity [54]. However, this stage was less frequently presented in the current sample. Our findings reflect and highlight the critical need to support osteosarcoma survivors in their search for meaning after the disruption of identity within cancer and cradle the quest narrative [11, 55]. More importantly, this study argues a distinction between personal and medical understandings of “the ending of cancer” [56, 57]. The process of narrating osteosarcoma survivorship experiences offers an alternative “ending” (see Table 8), allowing survivors to reestablish a sense of agency and document their personal transformation from chaos to a renewed sense of self‐identity in the recovery [57, 58, 59, 60]. This study demonstrates that Frank's three narrative types provide a valuable framework for understanding the lived experiences of osteosarcoma survivors, revealing that they are not passive recipients of their disease but active agents in their own recovery [42, 49].

This examination of osteosarcoma survivors' narratives within the Chinese context revealed that survivors experienced significant challenges in navigating the initial phase of their illness, characterized by limited professional support (see Table 4). This lack of support is a potential barrier to survivors' ability to transition from chaotic experiences toward narratives of restitution and, ultimately, personal growth. These findings underscore the critical need for professional interventions that facilitate cancer survivors' exploration of their post‐cancer experiences, enabling them to construct meaningful narratives and foster personal growth [42].

### 4.1 Alternative Narratives: Beyond Frank's Framework

Despite Frank's framework conceptualizing three narrative types that interpret cancer survivorship experiences within the Chinese context, we identified alternative narratives within osteosarcoma survivors that offer significant implications for psycho‐oncology practices.

First, regarding transitional narratives. We highlight the barriers to help‐seeking among osteosarcoma survivors. The results indicated a narrative plot about survivors who tend to reject the support offered by professional organizations or school teachers. We identified this narration associated with low help‐seeking willingness initially emerging in the transition from the medical context to regular daily life (Participants 4 and 12). As Holland et al. [61] argue, low willingness to psychosocial care among cancer survivors is related to a poor referral process from clinicians to the community and is usually associated with lower levels of illness knowledge and more avoidant coping styles. Consistent with Plage et al. [16], it is suggested that there is a critical need to facilitate the integration between medical and educational settings to support the developmental needs of youth cancer survivors and address the challenges of marginalization they may experience. Furthermore, these findings suggest that the illness narrative framework represents a nonlinear and complex process rather than a sequential progression. In this view, cancer care demands the integration of stigma‐reduction strategies and psychological practices.

Second, regarding consideration of culturally specific narratives. Specifically, we identified the dilemma of psycho‐oncology practices in the Chinese context. A narrative plot indicated that Chinese nurses might take accountability for offering counseling services to cancer patients and survivors (Participant 5). This narrative echoed previous studies that Chinese nurses indeed take predominant responsibility but with unsubstantial training for implementing psychological care for cancer survivors [62]. This finding is critical and might reflect the overwhelming work of nurses and the underdeveloped psycho‐oncology practices in China.

### 4.2 Rethinking Medical Discourse

While medical treatment is the most significant and effective way to treat osteosarcoma, limb salvage or other surgeries cause physical body changes, leading adolescents with osteosarcoma to suffer from stress, marginalization, and stigmatization [63, 64, 65].

As Blaxter [66] argues, the diagnosis of cancer relates to the sense of isolation. The medical diagnosis and doctors' treatment take the dominant position that overlooked the psychological needs of cancer survivors, which violates the patient‐centered approach (see Chaos Narratives). Similarly, Plage [11] underscores recognizing the disparity between cancer survivorship experiences and medicalized conceptualizations of cancer, which could provide a critical starting point for constructing meaning throughout the cancer journey. Aligning with Blaxter and Plage's perspective, we argue that exploring cancer survivors' narratives serves as a crucial complement to medical approaches. A narrative approach helps researchers dive into the world of osteosarcoma patients and survivors to explore their psychological needs. These results facilitate our understanding of sparkling moments and meanings beyond the illness [58, 67, 68]. This study emphasized the narrative plot of osteosarcoma survivors' journeys toward survival, focusing on how reflections on interpersonal relationships and the generation of positive personal growth constitute a significant aspect of meaning‐making beyond clinical treatment outcomes [42, 58, 67]. We echoed Coll‐Planas and Visa [67] that cancer survivors have tailor‐made narration to their survivorship. Furthermore, this narrative approach holds significant value, suggesting a potential for the sociological integration of clinical and psychological oncology practice that extends beyond the prevailing focus on conventional cancer treatments [58].

In this view, we respond to the notion that the exploration of cancer survivors' narratives is a “makeover” that sheltered the effect of cancer and the limits of the human body [69]. This study posits that coping with cancer cannot be adequately understood through dichotomous approaches. Instead, it emphasizes the value of survivor narratives as an alternative framework that reveals meaningful experiences beyond the disease and explores their potential influence on other individuals [58, 63].

### 4.3 Narratives and Cancer Survivors Empowerment

A progression from chaos to quest narratives was evident in the data, with 11 narratives demonstrating elements of orderly restitution narratives, while reflective segments within these narratives aligned with the characteristics of quest narratives [70]. While osteosarcoma can significantly disempower survivors [5, 71], our findings highlighted the transformative potential of the cancer experience, particularly within the quest narratives. Through retelling survivorship experiences, we documented survivors' quest narratives with regard to their adjustment, acquiring new knowledge, and developing crucial coping skills [5, 72]. These findings pointed out the empowering nature of narrative construction for osteosarcoma survivors, enabling them to build up their toolkit [60]. Furthermore, we suggest a potential empowerment approach which is narrative‐based practices, particularly collective narrative practices, to facilitate empowerment among this marginalized population [73, 74]. Collective narrative practices employ strategies to incorporate personal narratives into collective narratives that strengthen cancer survivors' power to resist cancer. These collective narratives manifest in diverse forms of documentation, such as the “Tree of Life,” which can serve as powerful tools for resistance.

### 4.4 Implication for Practices

Our exploration of survivors' narratives reveals their limited chance to accept official psychological intervention during the osteosarcoma treatment and recovery journey. Echoing the existing practices within the Chinese context, we argue that osteosarcoma survivors, and even other cancer survivors, might experience limited comprehensive psychological and developmental transition support. It implies a further reflection on the latent reasons that limit the expansion of psycho‐oncology support within the Chinese context, such as public awareness and insufficient policy support [75, 76]. Future studies suggest articulating the potential barriers to the integration of medical treatment and psychological support for osteosarcoma and other cancer survivors [33, 41, 76, 77].

The findings of this study echo the emerging trend that cancer caregivers use narrative approaches to help cancer survivors explore and document their journey [50, 78]. Considering narrative approaches not only share meaningful counter‐narratives beyond illness [40, 79, 80] but also provide potential practice integration with narrative therapy [50, 77]. It reminded practitioners that cancer survivors have a unique story waiting for documentation and blossoming [81, 82].

While evidence suggests that narrative approaches can effectively address the psychological needs of cancer survivors [50], their implementation within the context of busy clinical settings presents a significant challenge for nurses [83]. From this perspective, we imply the critique of solely medicalized psychological support, which reinforces dominant discourses and neglects the broader psychosocial needs of survivors [77, 84]. Given the distinct focus on medical and psychological practices among cancer survivors, we suggest that careful consideration of resource allocation between these disciplines within the Chinese context.

### 4.5 Limitation

We consider several limitations and critical reflections that emerged from this study. First, our sample encompasses a broad range of survivorship periods (i.e., 1–26 years) and a small sample size (i.e., 12 participants) representing diverse survivorship experiences. This wide temporal scope and small size may have obscured subtle narrative nuances and depth among participants. Second, the application of the Western illness narrative framework to Chinese osteosarcoma survivors' experiences may inadequately capture culturally specific family dynamics and interpersonal relationship changes during the cancer journey. Future research should aim to enhance illness narrative frameworks by incorporating cultural elements specific to cancer survivorship within Chinese contexts.

## 5 Conclusion

This study employed a narrative approach to investigate the experiences of Chinese osteosarcoma survivors, identifying three distinct narrative types: “No one knows: chaos,” “Rethinking: restitution,” and “Restarting and retelling: quest.” Narrative analysis revealed how these narratives reflect the survivors' journey from unpredictability and chaos toward the integration of meaning with coping strategies. While these narratives provided a valuable framework, we also identified alternative narratives that emerged beyond these established categories. These findings highlight the need to critically examine the current medical discourse and psycho‐oncology practice within the Chinese context. Moreover, we propose that narrative approaches can serve as an empowering framework for osteosarcoma survivors, fostering personal agency and facilitating identity reconfiguration. Our findings emphasize the critical need for integrating psychological support as a compulsory supplement into standard cancer care practices in China. Future research should expand exploration into the nuanced experiences of Chinese cancer survivors, exploring the specific cultural and social factors that may impact their survivorship journeys. This includes investigating barriers to the integration of medical treatment and psychological support within the cancer care system and exploring the perspectives of oncology caregivers to gain a more comprehensive understanding of the needs of this population.

## Author Contributions

**Ziqi Peng:** conceptualization, methodology, software, data curation, investigation, validation, formal analysis, resources, writing – original draft, writing – review and editing, project administration. **Suet Lin Hung:** validation, supervision, writing – review and editing, formal analysis. **Kwok Kin Fung:** methodology, validation, writing – review and editing, formal analysis, supervision.

## Ethics Statement

The design of this study went through ethical research approval by the Research Ethics Committee (REC) of the Faculty of Arts and Social Sciences at Hong Kong Baptist University. All the participants knew the study details and signed informed consent forms.

## Conflicts of Interest

The authors declare no conflicts of interest.

## Data Availability

The data that support the findings of this study are available from the corresponding author upon reasonable request.
